# Effect of Thermal Cycles on the Compressive Properties of 3D-Printed Polymeric Lattice-Reinforced Cement-Based Materials

**DOI:** 10.3390/polym17152137

**Published:** 2025-08-04

**Authors:** Can Tang, Yujie Zhou, Jing Qiao, Humaira Kanwal, Guoqian Song, Wenfeng Hao

**Affiliations:** 1 College of Civil Science and Engineering, Yangzhou University, Yangzhou 225127, China; 2 Faculty of Civil Engineering and Mechanics, Jiangsu University, Zhenjiang 212013, China; 3 College of Mechanical Engineering, Yangzhou University, Yangzhou 225127, China

**Keywords:** 3D-printed lattice-reinforced cement-based composites, compressive properties, thermal cycles, digital image correlation (DIC), acoustic emission (AE)

## Abstract

Existing studies have shown that placing 3D-printed lattices in cement matrices can effectively improve the ductility of cement-based composites. However, the influence of thermal fatigue effect on the mechanical properties of 3D-printed lattice-reinforced cement-based composites during service remains to be studied. In this paper, cement-based materials without lattices were used as the control group, and the uniaxial compressive mechanical properties of 3D-printed lattice-reinforced cement-based composites after thermal fatigue treatment under a temperature difference of 60 °C were tested. The number of thermal fatigue cycles was set to 45, 90, and 145 times, respectively. During the test, two non-destructive testing technologies, AE and DIC, were used to analyze the strength degradation and deformation law of 3D-printed lattice-reinforced cement-based composites with the increase in cycles. AE adopted the threshold triggering mode, and the channel threshold was 100 mv. The experiment showed that the compressive strength of the control group after 45, 90, and 145 thermal cycles decreased to 72.47% and 49.44% of that of the specimen after 45 thermal cycles, respectively. The strength of RO lattices decreased to 91.07% and 82.14% of that of the specimen after 45 thermal cycles, respectively, while the strength of SO lattices decreased to 83.27% and 77.96% of that of the specimen after 45 thermal cycles, respectively. The compressive strengths of the two types of lattices were higher than that of the control group after three cycles, indicating that 3D-printed lattices can effectively mitigate the influence of environmental thermal fatigue on the mechanical properties of cement-based materials.

## 1. Introduction

The service environment of concrete structures is subject to constant fluctuations. For example, under solar radiation, the exterior surfaces of buildings can reach significantly high temperatures. These surfaces then rapidly cool due to environmental factors such as wind, cold currents, rain, or hail. The repeated impact of this rapid heating and cooling cycle induces environmental thermal fatigue in structural concrete characterized by alternating cold and hot conditions [1,2,3,4]. This type of thermal stress leads to damage and cracking in high-performance concrete, significantly reducing its resistance to permeability. These effects compromise the integrity and durability of concrete structures and notably hasten deterioration processes such as salt corrosion, freeze–thaw cycles, and moisture variations, further impairing their durability. As a quasi-brittle material, concrete exhibits inherent brittleness, low tensile strength, and susceptibility to brittle failure. The significant impact of environmental thermal fatigue on concrete’s performance underscores the importance of investigating the mechanical properties and evolutionary trends of 3D-printed cement-based composites subjected to these conditions [5,6].

Kraft et al. [7] discovered that cement paste, mortar, and concrete each have significantly different coefficients of thermal expansion. Given that aggregates constitute the majority of concrete’s volume, they play a pivotal role in determining its thermal expansion coefficient. Baluch [8] proposed that during thermal cycling, discrepancies in the thermal expansion coefficients between the matrix and aggregate lead to uncoordinated deformation, generating tensile stress at the interfacial transition zone. When this stress surpasses the ultimate strength of the zone, microcracks form, impairing the mechanical properties of concrete. Lin et al. [9] investigated the effects of temperature cycling from 20 °C to 145 °C on the mechanical properties of cement-based materials, finding that both peak stress and elastic modulus decreased with an increasing number of cycles, detrimentally affecting the macroscopic properties. Kanellopoulos et al. [10] examined the effect of temperature cycling between 20 °C and 90 °C on the fracture energy of concrete and observed more than a 10% decrease in fracture energy after 90 cycles, largely attributed to microcrack propagation and spreading between the coarse aggregate and matrix. Furthermore, Mahboub et al. [11] used Ansys 8.0 software to simulate the behavior of road concrete under a temperature field, suggesting that the alternating high and low temperatures exerted a more significant impact on road concrete than vehicular load. Additionally, Wei et al. [12] explored the influence of temperature fluctuations on early stress development in confined concrete and introduced an enhanced MPS model to predict early tensile stress in concrete, incorporating temperature’s effect on creep.

Numerous scholars have demonstrated that environmental thermal fatigue can result in uncoordinated deformation within concrete, arising from temperature gradients and the disparate thermal properties of its constituents. This misaligned deformation leads to damage and cracking in the material. The existing body of research into the thermal properties of concrete materials, along with the varying behaviors of constituent phases and non-uniform deformation at internal interfaces, lays a foundational framework for investigating the performance evolution mechanisms of 3D-printed cement-based composites under environmental thermal fatigue.

In recent years, extensive research has been conducted on the impact of 3D-printed polymer lattices on the compressive mechanical properties of cement-based composites [13]. Zeng et al. [14] developed a novel form of 3D-printed continuous fiber reinforced thermoplastic polymers (CFRTPs) reinforcement for 3D-printed concrete structures, and the tensile behavior of 3D-printed CFRTP bars and grids was conducted. Then, the CFRTP reinforcement was used for 3D-printed high-performance concrete to explore the effectiveness of the reinforcement. Salazar et al. [15] investigated the mechanical properties of ultra-high-performance concrete reinforced with 3D-printed polymer lattices under uniaxial compression loads. Bogusz et al. [16] performed quasi-static axial compression tests on a three-dimensional lattice created using stereolithography (SLA), studying the compression curves and deformation processes across five distinct lattice geometries. Tao et al. [17] utilized fused filament fabrication (FFF) technology to print two types of lattice structures, which were then infused with rigid polyurethane foam (RPUF) to fabricate structural composites. These composites demonstrated significantly enhanced elastic limits, compressive moduli, and energy absorption capacities compared to pure RPUF. Ghannadpour et al. [18] explored the compressive behavior of six pillar-based topologies printed through digital light processing (DLP) via both experimental tests and numerical simulations. Moreover, Tzortzinis et al. [19] introduced a novel methodology employing steel-assisted truss lattice reinforcements to contain concrete/mortar materials, observing that the auxiliary lattice provided superior confinement to the mortar matrix under axial compression compared to traditional mortar samples. While research on 3D-printed lattice-reinforced cement-based composites has primarily concentrated on their mechanical properties at room temperature, the influence of environmental thermal fatigue on these properties remains an important area of study.

In this study, two polymer lattices with differing structures were fabricated using multi-jet fusion (MJF) technology [20,21,22,23], ensuring an identical volume for each configuration by design. These lattices were then integrated into molds to cast the cement matrices, forming lattice-reinforced cement-based samples. Following a standard 28-day curing period, the samples underwent thermal fatigue treatment, experiencing temperature fluctuations of 60 °C over 45, 90, and 145 cycles. Subsequent to this treatment, uniaxial compression tests were performed to assess alterations in mechanical properties [24,25,26,27]. The deterioration in strength and deformation of the 3D-printed lattice-reinforced cement-based composites were quantitatively analyzed through nondestructive acoustic emission (AE) and digital image correlation (DIC) testing techniques, correlating the findings with the number of thermal cycles experienced. This study advances the field by focusing on RO and SO lattices under thermal fatigue, a context unexamined in prior lattice research. Addressing the critical gap in understanding how 3D-printed lattices modulate thermal fatigue damage in cement composites, whereas earlier work focused on conventional concrete or room-temperature lattice performance. Integrating AE and DIC to unravel the mechanistic links between lattice topology, thermal cycling, and deterioration—providing a more nuanced understanding than traditional mechanical testing alone.

## 2. Sample Design

Two distinct 3D-printed lattice geometries, the rhombicuboctahedron (RO) and the strengthened octagonal (SO), were utilized to reinforce cementitious composites [28]. For comparative analysis, a plain cementitious specimen without lattice reinforcement served as the control.

### 2.1. Lattice Design

In this study, a 3D-printed lattice was fabricated using multi-jet fusion (MJF) technology with PA6 as the raw material. This study employed a controlled variable method to design lattice structures, maintaining identical volume fractions for two different configurations to investigate the mechanical performance differences between plain cement mortar and the lattice structures under environmental thermal fatigue. The specific design procedure was conducted as follows: First, the strut diameter of unit cells was precisely adjusted to control the lattice volume, ensuring consistent volume fractions for different lattice configurations within the cement mortar matrix. A quantitative relationship model between strut diameter and lattice volume was established using the information query function of CAD modeling platform. By systematically varying the strut diameter parameters (*d_i_*, *i* = 2–7), the lattices were replicated and assembled using AutoCAD 2023 software, with their corresponding volumes calculated. Finally, the mathematical relationship between diameter variables and lattice volume (*V*) was fitted to a cubic polynomial (1) and (2) using the least squares method.

(1) $$V_{\mathrm{RO}}=f\left(d_{1}\right)=-2\times 10^{-5}d_{1}+8115.9d_{1}^{2}-1151.5d_{1}^{3}$$

(2) $$V_{SO}=f\left(d_{2}\right)=0.0003d_{2}+7072.3d_{2}^{2}-900.55d_{2}^{3}$$

The cement matrix specimens were fabricated in standard cubic dimensions of 42 mm × 42 mm × 42 mm, yielding a total volume of 74,088 mm^3^. The volume fraction of lattice structures within the cement matrix was controlled at 8% according to the experimental design. Based on the computational model established by Equations (1) and (2), the strut diameter parameters for two distinct lattice configurations were determined, with the calculated unit cell strut diameters presented in detail in Table 1.

### 2.2. Cement-Based Materials

The dimensions of the uniaxial compression samples were 42 mm × 42 mm × 42 mm. The samples were prepared using raw materials that included cement, fly ash, standard sand, a water reducer, and water. The main performance indicators for these raw materials are as follows:

(1) Cement: ordinary Portland cement P·O 42.5 was produced by Jiangsu Hailin Cement Co., Ltd.(Taizhou, China), and its main mineral composition is shown in Table 2. The particle size distribution was concentrated between 3 μm and 60 μm.
(2) Standard sand: Chinese ISO standard sand was produced by Xiamen ISO Standard Sand Co., Ltd. (Xiamen, China) Its main mineral composition is shown in Table 3. To ensure that the mortar had sufficient fluidity during sample preparation, the standard sand was screened.
(3) Fly ash: The first-class fly ash was produced by Gongyi Bairun Refractory Co., Ltd. (Zhengzhou, China), and its main chemical composition is shown in Table 4.
(4) Water-reducing agent: Polycarboxylic acid water-reducing agent was produced by Fokker Technology (Suzhou) Co., Ltd. (Suzhou, China), and its performance indices are shown in Table 5.

**Figure 1:**
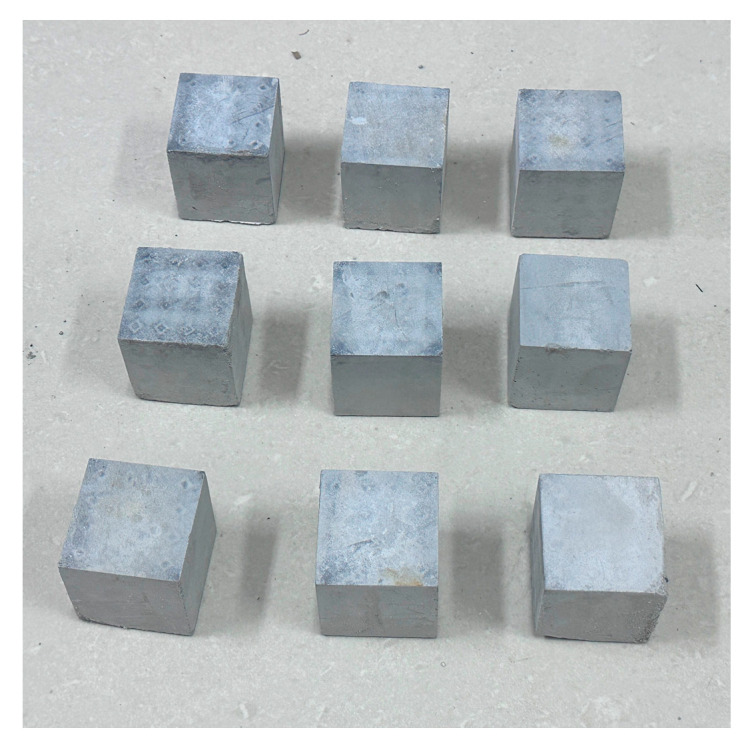
D-printed lattice-reinforced cement-based samples.

The samples after standard curing for 28 days are shown in Figure 1.

## 3. Experimental Design

**Figure 2:**
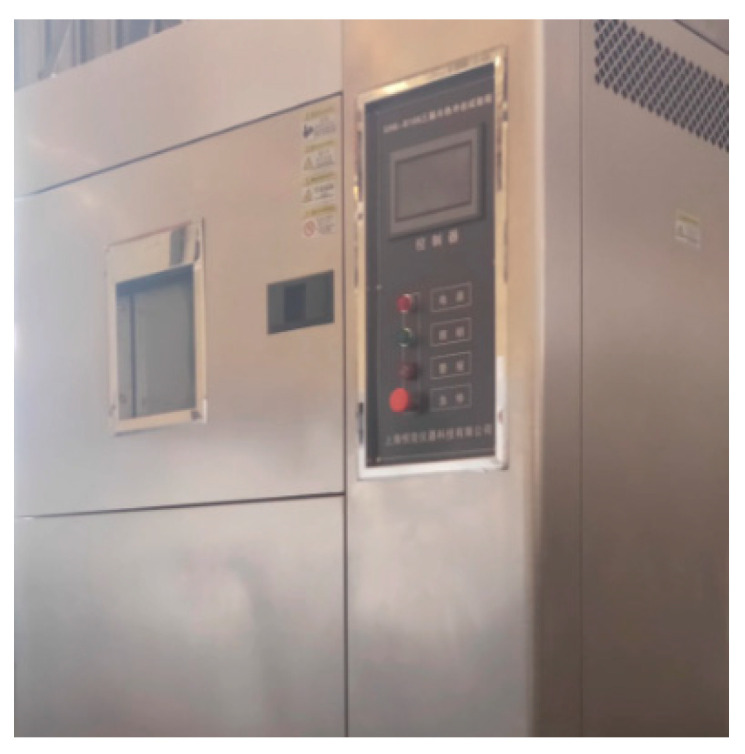
High-temperature test chamber.

Following a standard curing period of 28 days, the cement-based composite material samples were allowed to acclimate in a room for one week to ensure complete evaporation of surface moisture. Subsequently, these samples were placed in a high-temperature test chamber, as depicted in Figure 2 High-temperature test chamber (model SET-Z-041LX) (Aspec Environmental Instruments (Shanghai) Co., Ltd., Shanghai, China.). The environment within the control box was pre-programmed.

**Figure 3:**
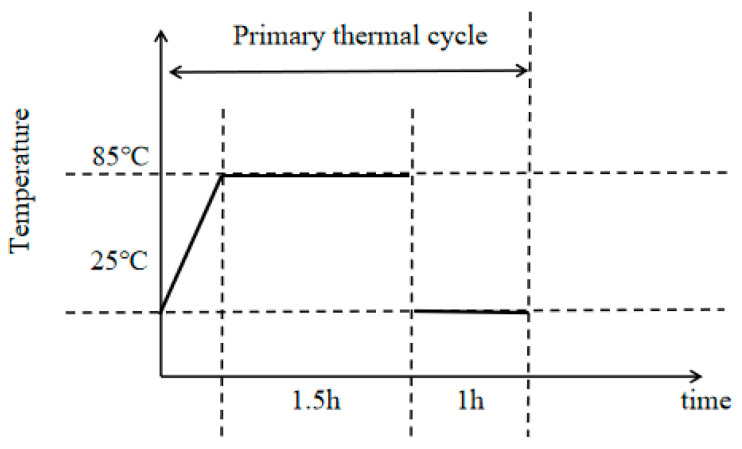
Schematic diagram showing the heat cycling mechanism.

During the heating process, the heating rate was maintained at or below 3 °C/min to mitigate thermal shock effects on the sample, as detailed in references [30,31,32,33]. For this experiment, a heating rate of 2 °C/min was employed to raise the temperature in the high-temperature chamber from room temperature to the target of 85 °C. The temperature was then maintained at 85 °C for 1.5 h before being reduced back to room temperature over a period of 1 h. This procedure constituted one thermal cycle, as depicted in Figure 3 The samples underwent 45, 90, and 145 thermal cycles.

**Figure 4:**
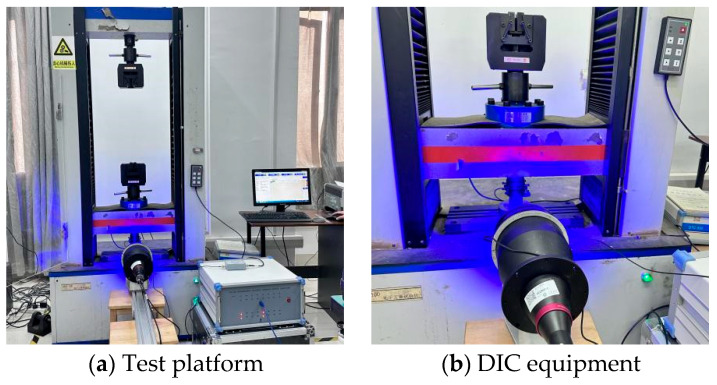
Experimental equipment.

The compression test setup is illustrated in Figure 4. A universal testing instrument, DNS-100 (Changchun Institute of Mechanical Science Co., Ltd., Changchun, China), capable of handling loads up to 100 kN, was selected to perform quasi-static loading at a rate of 1 mm/min. Uniaxial compression tests were conducted on 3D-printed lattice-reinforced cement-based composite samples. AE technology monitored specimen damage during the loading process, while DIC tracked deformation across the entire specimen.

During the loading process, the DS2-8A (four-channel) full-information AE signal analyzer produced by Ruandao Company (Beijing, China) was used for the collection and analysis of AE signals. The diameter of the sensor is 16 mm. The sensor is connected to the AE signal analyzer through a 40 dB intelligent AE preamplifier. The experimental acquisition accuracy is 16 bits, the sampling rate is 3 M, and the sampling time interval is 0.3333 μs. The threshold triggering mode is adopted. According to existing studies [34], the channel threshold for 3D-printed cement-based composites is set to 100 mV.

The image acquisition system comprised a cold light source, a BT-23120 telephoto lens, an MV-EM510 M/C CCD camera, and a supporting bracket(provided by Wilkesh Digital Image Technology Co., Ltd. (Beijing, China)). The telecentric lens featured a magnification of 0.072×. The CCD camera boasted a resolution of 2456 × 2058 pixels, amounting to a total of approximately 5 million pixels. Image acquisition software provided by Wilkesh Digital Image Technology Co., Ltd. (Beijing, China), facilitated data capture at a rate of 8 frames per second. A 2D-DIC system from CSI Company (Beijing, China)was utilized for processing the images throughout the entire loading process.

## 4. Results and Discussion

In this paper, uniaxial compression tests were conducted on cement-based composites reinforced with three types of lattices: no lattice, RO lattice, and SO lattice. Each type consisted of 9 samples, totaling 27 samples. These were evenly distributed into three groups, each subjected to 45, 90, and 145 environmental thermal fatigue cycles, respectively. Following treatment, the samples underwent uniaxial compression loading. For further analysis, the data group representing the highest peak load within each category was selected for AE characteristics analysis and strain field analysis.

### 4.1. Stress–Strain Curve Analysis

**Figure 5:**
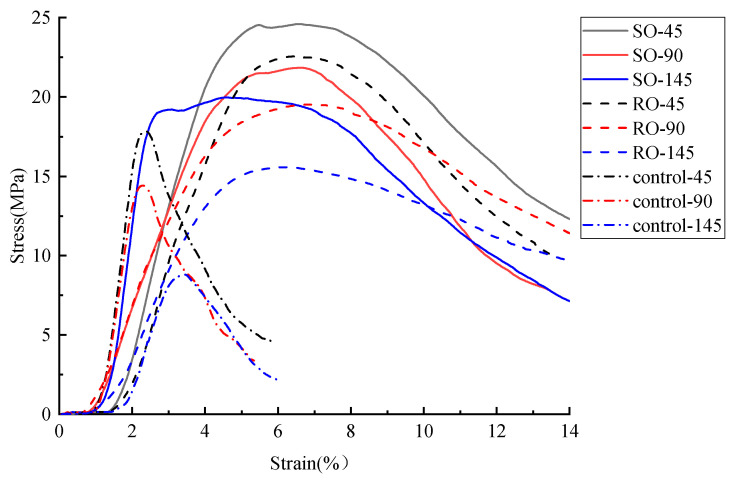
Stress–strain curves of lattice-reinforced cement-based composites under different numbers of thermal fatigue cycles.

**Figure 6:**
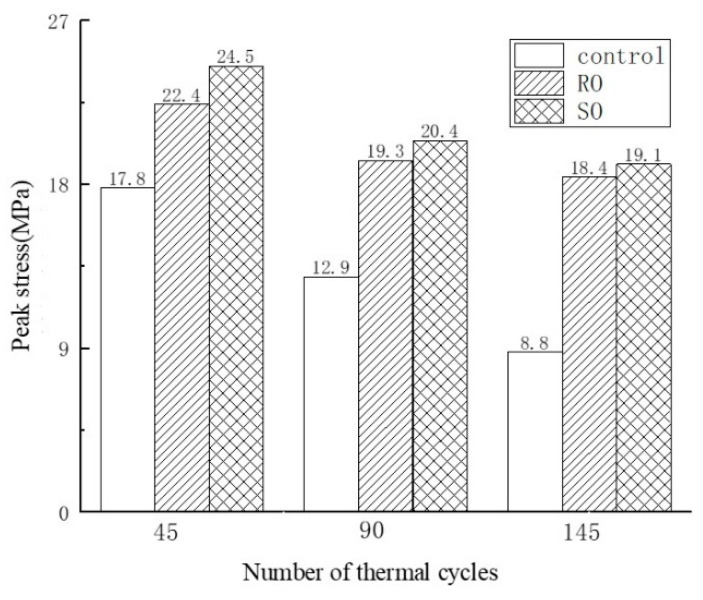
The peak load of lattice-reinforced cement-based composites under different numbers of thermal fatigue cycles.

The stress–strain curves of cement-based composite specimens after undergoing various thermal cycles are displayed in Figure 5 and Figure 6 shows that after 45, 90, and 145 thermal cycles, the peak loads for the non-reinforced cement-based composites were 17.8 MPa, 12.9 MPa, and 8.8 MPa, respectively. The strength of these specimens diminished to 72.47% and 49.44% of the initial value after 45 and 90 thermal cycles, respectively. For the RO lattice-reinforced samples, the peak loads were 22.4 MPa, 19.3 MPa, and 18.4 MPa, showing a decrease in strength to 91.07% and 82.14% after 45 and 90 cycles, respectively. Similarly, the SO lattice-reinforced samples exhibited peak loads of 24.5 MPa, 20.4 MPa, and 19.1 MPa, with their strengths declining to 83.27% and 77.96% after the same number of cycles. Notably, the specimens reinforced with 3D-printed lattices experienced significantly less reduction in compressive strength across the same number of cycles compared to those without lattice reinforcement.

### 4.2. AE Signal Analysis

**Figure 7:**
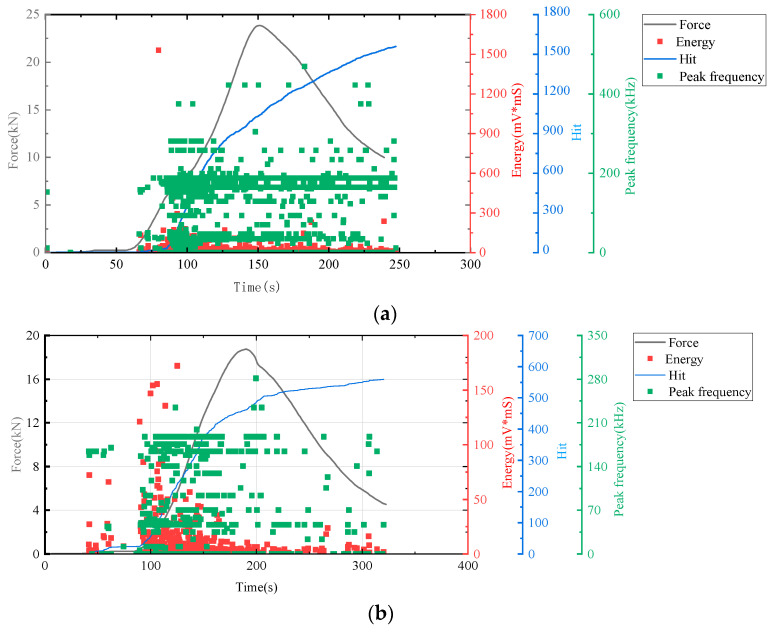

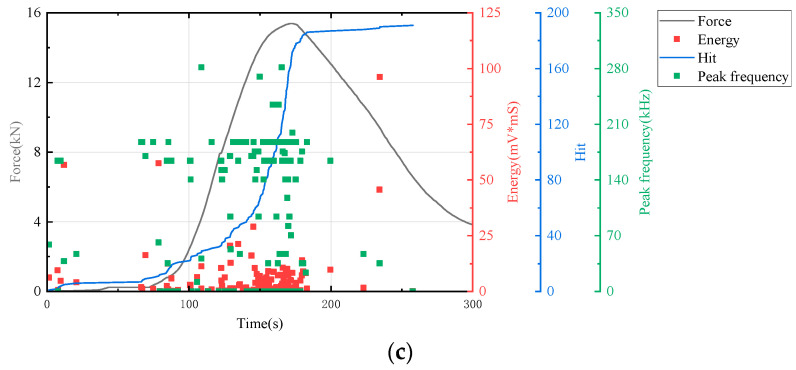
AE and stress–strain curves of cement-based samples without lattice reinforcement. (**a**) Cement-based samples without lattice reinforcement after 45 thermal cycles. (**b**) Cement-based samples without lattice reinforcement after 90 thermal cycles. (**c**) cement-based samples without lattice reinforcement after 145 thermal cycles.

**Figure 8:**
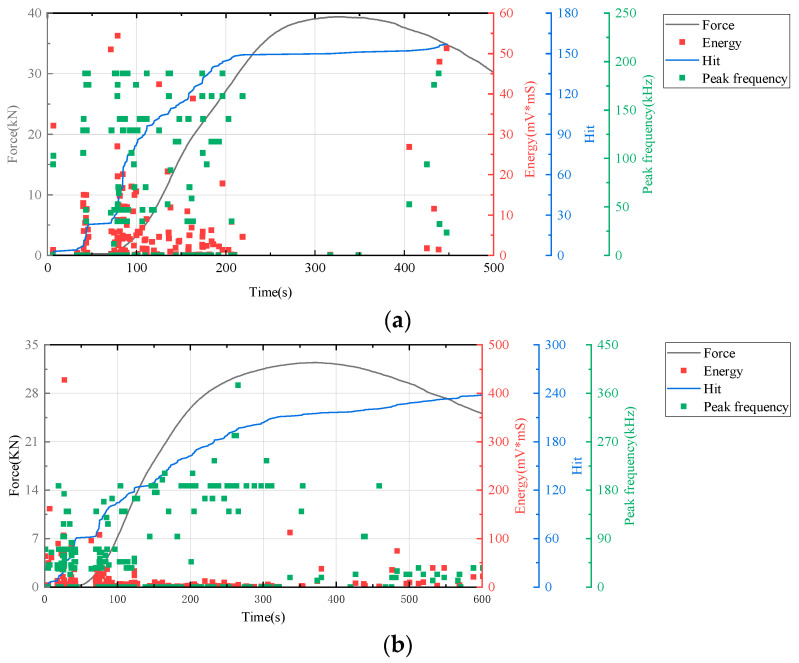

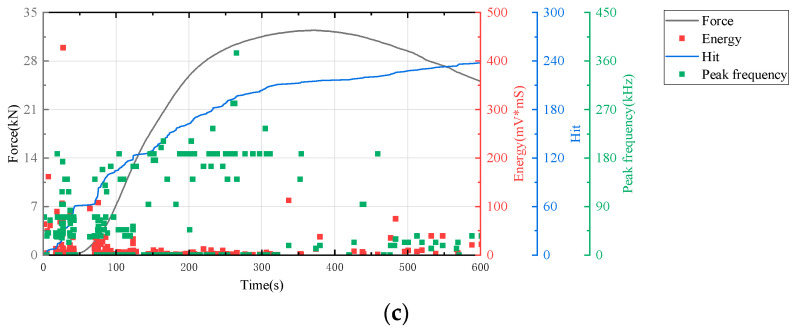
AE and stress–strain images of cement-based samples with RO lattice reinforcement. (**a**) Cement-based samples with RO lattice reinforcement after 45 thermal cycles. (**b**) Cement-based samples with RO lattice reinforcement after 90 thermal cycles. (**c**) Cement-based samples with RO lattice reinforcement after 145 thermal cycles.

**Figure 9:**
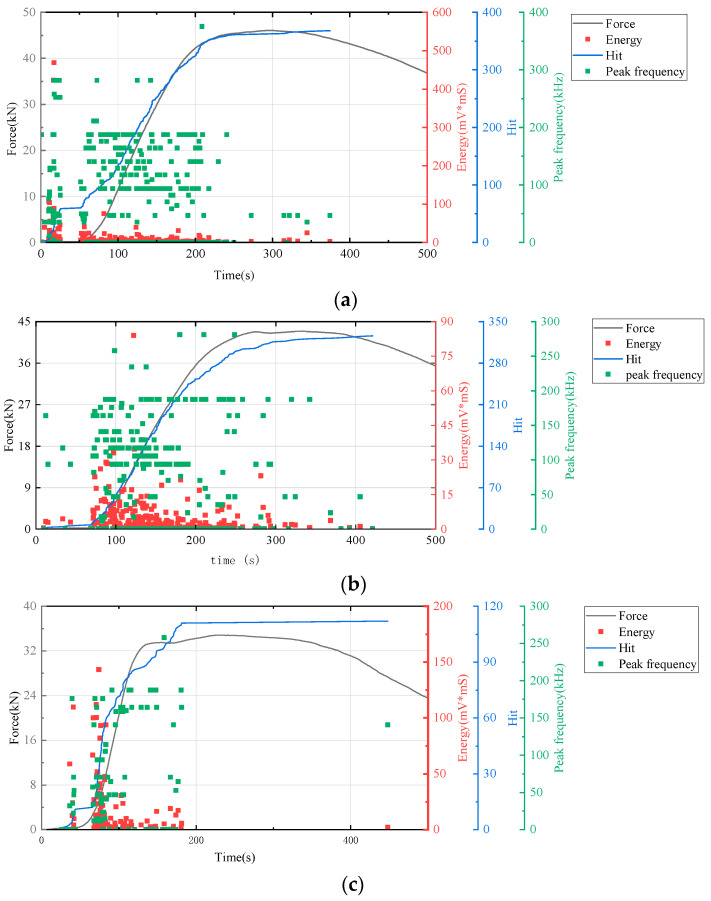
Acoustic emission and stress–strain curves of cement-based samples with SO lattice reinforcement. (**a**) Cement-based samples with SO lattice reinforcement after 45 thermal cycles. (**b**) Cement-based samples with SO lattice reinforcement after 90 thermal cycles. (**c**) Cement-based samples with SO lattice reinforcement after 145 thermal cycles.

In this study, AE technology was employed to monitor real-time signals such as deformation and fracture in materials or structures under load, a process driven by the strain energy released from stress waves. We utilized key AE parameters including hits, energy, and peak frequency as evaluation indicators to generate time–force, time–hit, time–energy, and time–peak frequency curves. Analysis of these curves enabled the assessment of the mechanical responses and deformation characteristics of cement-based samples under compression. The AE characteristics of the samples during loading were further analyzed by correlating the AE parameters with the stress–strain curves depicted in Figure 7, Figure 8 and Figure 9.

Figure 7, the lattice-unreinforced cement-based control sample initially exhibited elastic deformation. With rapidly increasing loads, the AE hit rates markedly accelerated, leading to a progressive rise in cumulative impacts. As loading continued, the formation of further cracks in the composite resulted in a higher frequency of AE hits. During the plastic deformation phase, the specimen underwent irreversible changes due to the sustained load, leading to extensive internal damage. As loading persisted, this damage intensified, prompting a renewed acceleration in the rate of AE hits. Ultimately, when the specimen’s load-bearing capacity was surpassed, it experienced significant levels of damage, although to a lesser extent than in earlier stages, leading to a reduction in AE hits.

Figure 8 illustrates that the AE parameters of RO lattice-reinforced cement-based composites during uniaxial compression are comparable to those observed in non-lattice-reinforced cement-based specimens.

All results indicated an increase in the number of AE hits during the initial stage of cement matrix compression. Throughout this stage, changes in energy and peak frequency were relatively minor.

The time–force curve (in Figure 9) shows that the 3D-printed lattice delayed the failure of the sample. When combined with the time–hit curve from AE data, it is evident that the number of hits for RO and SO lattice-reinforced cement-based samples plateaued during the initial loading phase. This suggests that the lattice structure in these samples significantly alters the failure mode of cement-based composites under uniaxial compression, effectively diminishing material damage. For lattice-reinforced specimens, this characteristic remained consistent despite an increase in the number of thermal cycles. However, the accumulated number of impacts reveals that a higher number of thermal cycles exacerbates material damage in the initial compression stage.

### 4.3. DIC Signal Analysis

In this study, deformation images of cement-based composite samples were captured throughout the loading process and analyzed using VIC-2D 6 (Beijing Ruituo Shichuang Technology Co., Ltd. Beijing China) to obtain strain maps after various thermal cycles. These images revealed significant alterations in the horizontal strain field (exx), whereas the vertical strain field (eyy) showed minimal changes. This section focuses on analyzing the strain distribution within the horizontal strain field during uniaxial compression tests. By integrating the stress–strain properties of the samples at different stages with the time–force curves, DIC images corresponding to specific feature points were selected for detailed analysis.

**Figure 10:**
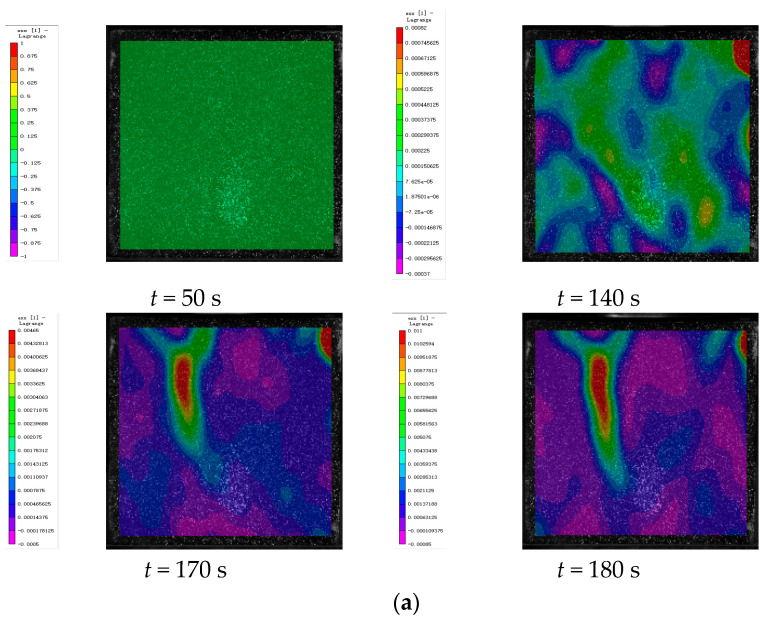

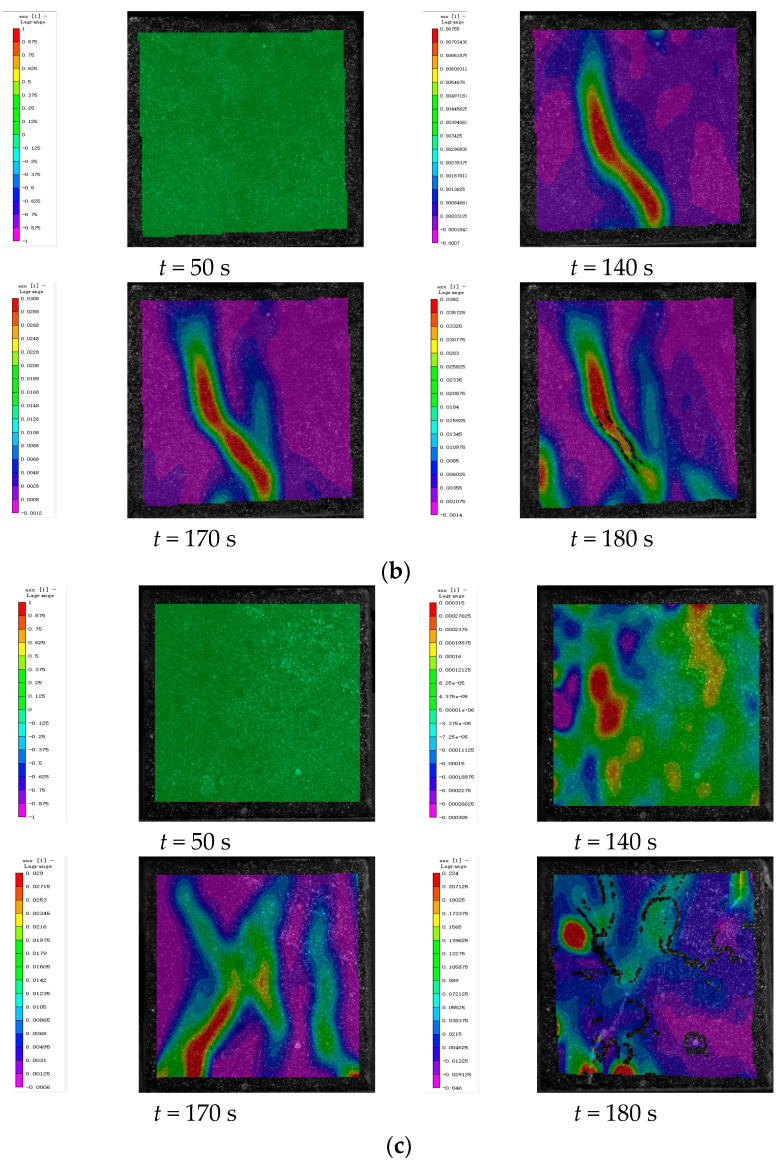
DIC monitoring strain cloud of cement-based composite without lattice reinforcement. (**a**) Cement-based composite without lattice reinforcement after 45 thermal cycles. (**b**) Cement-based composite without lattice reinforcement after 90 thermal cycles. (**c**) Cement-based composite without lattice reinforcement after 145 thermal cycles.

**Figure 11:**
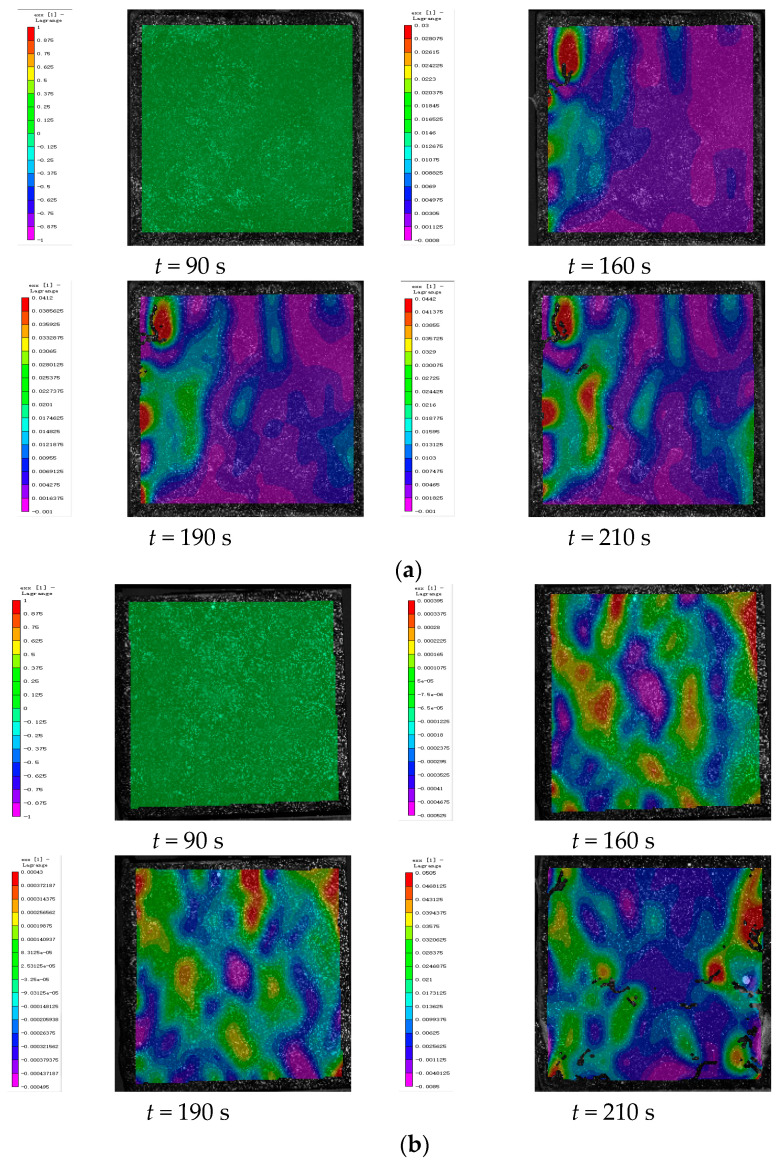

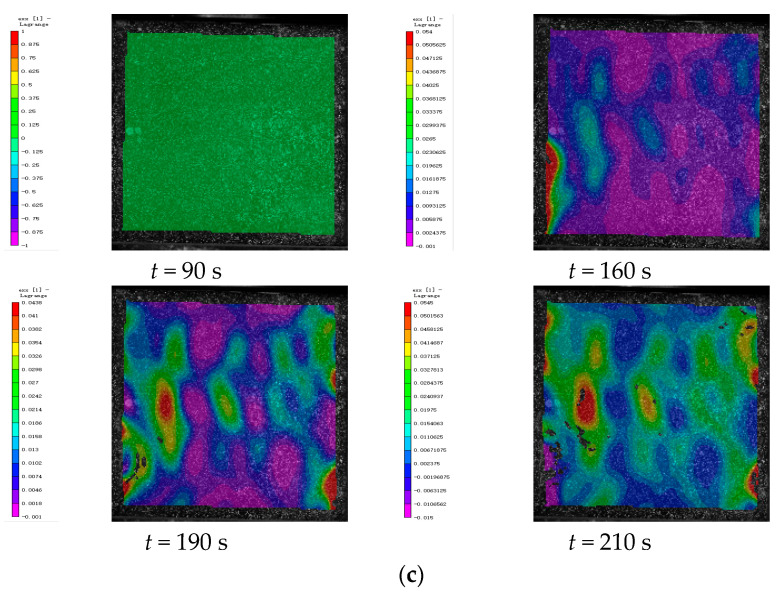
DIC monitoring strain cloud of cement-based composite with RO lattice reinforcement. (**a**) Cement-based composite with RO lattice reinforcement after 45 thermal cycles. (**b**) Cement-based composite with RO lattice reinforcement after 90 thermal cycles. (**c**) Cement-based composite with RO lattice reinforcement after 145 thermal cycles.

**Figure 12:**
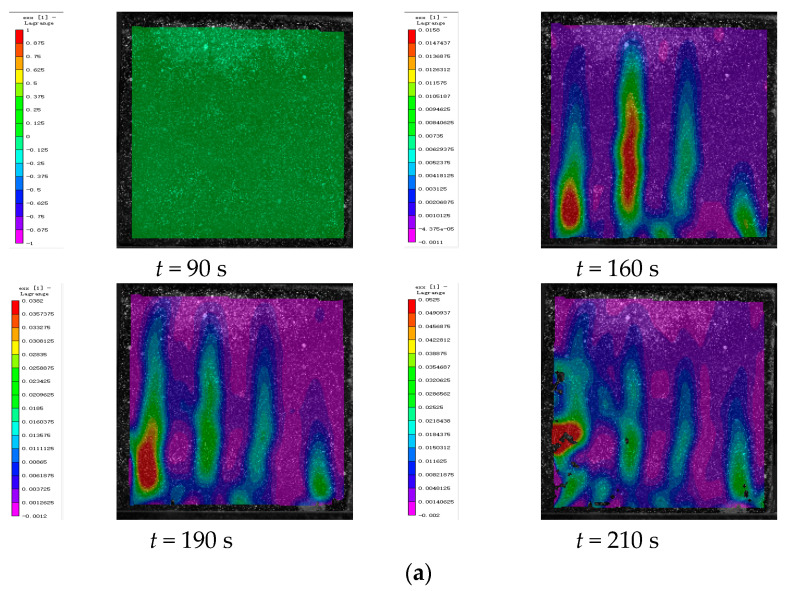

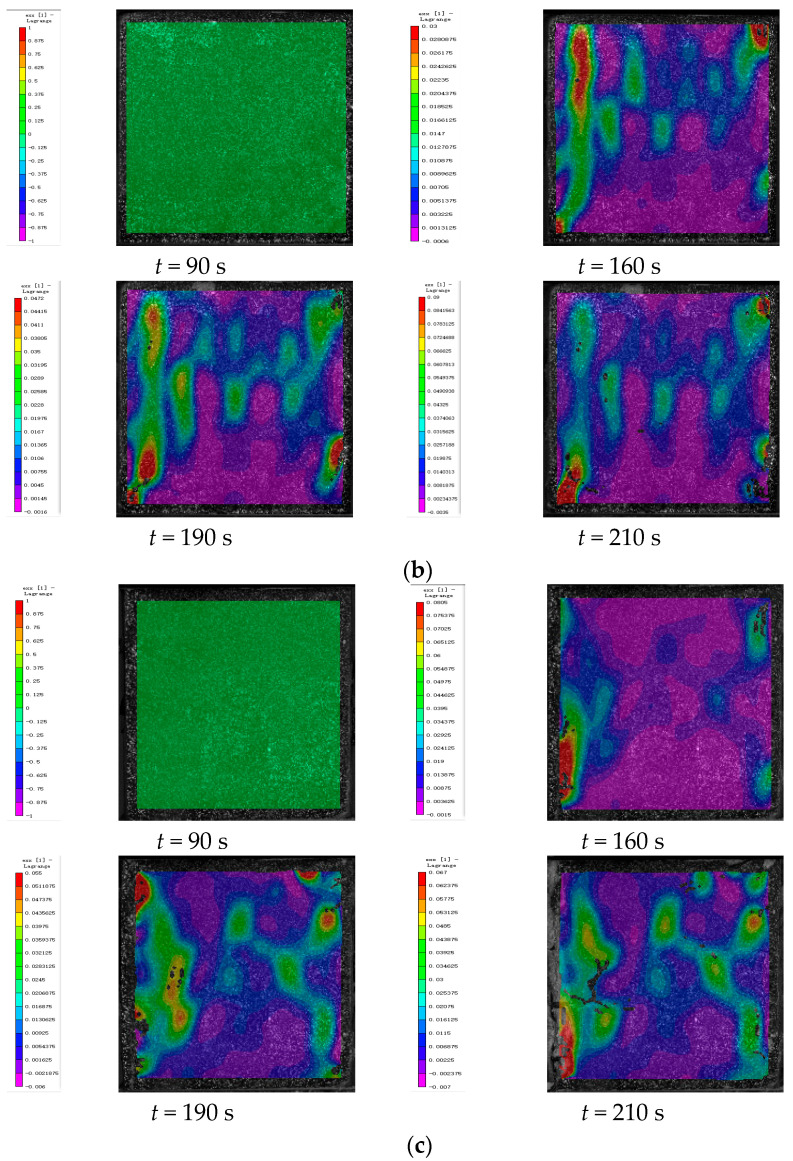
DIC monitoring strain cloud of cement-based composite with SO lattice reinforcement. (**a**) Cement-based composite with SO lattice reinforcement after 45 thermal cycles. (**b**) Cement-based composite with SO lattice reinforcement after 90 thermal cycles. (**c**) Cement-based composite with SO lattice reinforcement after 145 thermal cycles.

Figure 10, Figure 11 and Figure 12 show the strain maps for cement-based samples without lattice reinforcement subjected to varying thermal fatigue cycles at different time points. The analysis of these strain maps for the unreinforced cement-based material reveals that the area of strain within the sample expanded progressively with an increase in the number of thermal cycles. Notably, the sample failed at 145 thermal cycles. This observation indicates that continuous thermal cycling significantly diminishes the compressive properties of the material. Despite these changes in mechanical properties, the ambient temperature did not alter the brittleness of the material. Consequently, the deformation of the material primarily appeared as a large crack, resulting from significant localized strain.

Figure 11 illustrates the strain maps of 3D-printed lattice-reinforced cement-based samples subjected to varying numbers of environmental thermal fatigue cycles at different time points during uniaxial compression tests.

Figure 12 presents the strain maps of 3D-printed lattice-reinforced cement-based samples featuring SO structures, captured at various time points during uniaxial compression tests following 45, 90, and 145 environmental thermal fatigue cycles, respectively.

As can be seen from Table 6, for specimens of the same type under different numbers of thermal cycles, the maximum strain of the three types of specimens (control, RO, and SO) generally increases with the increase in the number of thermal fatigue cycles. This indicates that the lateral expansion deformation of the specimens becomes more obvious as the number of cycles accumulates, and also confirms that thermal fatigue cycles do promote material deformation. However, although the strain of all three types of specimens increases, the strain growth of RO and SO specimens with lattice reinforcement is much slower than that of the control group without lattice reinforcement. This intuitively reflects that the lattice can play a role in inhibiting deformation. Further analysis shows that thermal fatigue treatment does reduce the mechanical properties of 3D-printed lattice-reinforced cement-based composites, specifically in the form of increased strain and weakened load-bearing capacity. But compared with the control group of cement-based specimens without lattice reinforcement, the addition of 3D-printed lattices can significantly reduce the damage to the mechanical properties of cement-based materials caused by alternating high and low temperature environments by virtue of their own structural constraint effect.

By further comparing the specimens with two different lattice structures (RO and SO), the maximum strains of RO lattice after 45, 90, and 145 thermal fatigue cycles are 0.0442, 0.0515, and 0.0545, respectively, while the maximum strains of SO lattice under the same number of cycles reach 0.0525, 0.09, and 0.067, respectively. It can be seen from the data that the maximum strain of RO lattice is smaller than that of SO lattice under all three cycle numbers. Moreover, with the increase in the number of cycles, the strain of RO increases from 0.0442 to 0.0545, with an increase of approximately 23.3%; the strain of SO increases from 0.0525 to 0.067, with an increase of approximately 27.6%. This indicates that after the action of environmental thermal fatigue, RO lattice has a better inhibitory effect on lateral expansion deformation and stronger deformation resistance than SO lattice.

By comparing the DIC strain contour maps of RO and SO lattice-reinforced cement-based composites under different thermal cycle counts, it is evident that environmental thermal fatigue does not alter the stress redistribution effect of 3D-printed lattices on the specimens. Compared with cement-based composites without lattice reinforcement under the same number of cycles, the addition of lattices can effectively delay the cracking of the specimens.

### 4.4. Morphological Analysis

**Figure 13:**
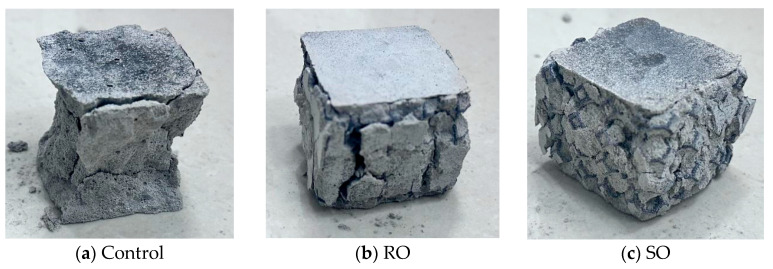
Failure morphology of cement-based samples reinforced by different lattice structures.

Following the uniaxial compression tests, the cement matrix that was peeled off the surface of the specimens revealed the failure morphology of cement-based specimens reinforced with various lattice structures, as presented in Figure 13. This observation suggests that the 3D-printed lattice influenced the phase composition of the cement matrix. During compression, this modification enabled the sample to withstand higher loads, thereby enhancing its compressive strength.

This study investigated the compressive mechanical properties of cement-based materials with lattice reinforcements of different structural forms under various thermal cycle conditions. The lattice structure was found to enhance the compressive load-bearing capacity of the materials, and deformation characteristics were analyzed, as detailed in Table 7. Sections (a)–(c) of Table 7 demonstrate that the cement-based samples lacking lattice reinforcements predominantly failed due to large cracks that compromised their load-bearing capacity. The deformation process involved friction between the sample and both the upper and lower indenters of the testing machine, leading to larger deformations at both surfaces of the sample due to external constraints, while the middle part contracted. This constrained deformation resulted in increased internal stress, ultimately causing the material to crack. Moreover, the data revealed that with an increasing number of thermal fatigue cycles, the compressive strength of the non-reinforced cement-based samples decreased, and the incidence of cracking in these samples increased.

Table 7d–i illustrates that for the cement-based composites with RO and SO lattice reinforcements, the lattice structure effectively constrained the cement matrix. Further, it redistributed the internal stresses through deformation, thereby enhancing the structure’s load-bearing capacity. This study compared the deformation characteristics of cement-based samples reinforced with an RO lattice after 45, 90, and 145 thermal cycles. With an increase in the number of thermal fatigue cycles, the constricting effect of the lattice on the cement matrix diminished, leading to an escalation in the extent of crack damage on the specimen surfaces. Although the compressive deformation characteristics of the SO lattice-reinforced samples were similar to those of the RO-reinforced samples, the RO lattice exhibited a stronger constraining impact on the cement matrix.

## 5. Conclusions

This study evaluated the compressive properties of 3D-printed lattice-reinforced cement-based composites subjected to thermal fatigue treatment at 60 °C, using lattice-free materials as a control. The composites underwent 45, 90, and 145 thermal fatigue cycles. We assessed the deterioration in strength and deformation of the 3D-printed lattice-reinforced composites through AE testing and DIC. Experimental findings indicated that the 3D-printed lattice mitigated the impact of environmental thermal fatigue on the mechanical properties of the cement-based materials. Some important conclusions are listed as follows:

(1) Based on the acoustic emission time–hit count curve, it can be seen that under the action of thermal cycles, the cement-based composites reinforced with RO and SO lattice structures can still change the failure mode under uniaxial compression, which is manifested by an obvious plateau in the number of hits at the initial loading stage. This effectively delays the failure under compression and reduces material damage, and this core function does not change with the increase in the number of thermal cycles. However, an increase in the number of thermal cycles will lead to an increase in the cumulative hit count of lattice-reinforced specimens at the initial compression stage; that is, it will aggravate the damage in their initial compaction stage.
(2) A comparison of strain cloud data of the control group and RO- and SO-lattice-reinforced cement-based composites under different thermal cycle counts reveals that an increase in thermal fatigue cycles exacerbates lateral expansion deformation in both the lattice-free control group and the RO- and SO-lattice-reinforced groups, indicating that thermal cycling, as a typical deterioration factor, increases material deformation and reduces load-bearing capacity. Although thermal cycling affects all three types of specimens, the deformation growth rate of the RO and SO lattice-reinforced groups is significantly lower than that of the control group, demonstrating that 3D-printed lattices can mitigate thermal cycle-induced damage through structural constraints and enhance thermal fatigue resistance. Under thermal cycling, the RO lattice-reinforced group consistently exhibits a lower deformation degree and a smaller deformation increase than the SO lattice-reinforced group, thus showing better performance in inhibiting lateral expansion deformation. Additionally, the stress redistribution effect of both lattices remains unaffected by thermal cycling, and they still effectively delay specimen cracking compared to the control group.
(3) Under the action of thermal cycles, there are obvious differences in the failure and deformation characteristics of different specimens. For cement-based specimens without lattice reinforcement, failure is mainly caused by large cracks that affect bearing capacity. During compression, due to the friction between the indenter and the specimen, they show a deformation pattern of “upper and lower parts constrained, middle part shrinking”, and stress is likely to accumulate inside, leading to cracking. Moreover, the more thermal cycles there are, the worse the bearing capacity becomes and the more severe the cracking gets. For RO and SO lattice-reinforced specimens, both can improve bearing capacity through lattice constraint and stress redistribution, with similar compression deformation characteristics. However, an increase in the number of thermal cycles will weaken their constraint effect and intensify crack propagation. Nevertheless, the initial constraint effect of RO lattices is better than that of SO lattices. Therefore, under the same number of thermal cycles, the constraint effect of RO lattices decays more slowly, and their anti-cracking performance is better.

## Figures and Tables

**Table 1:** Design parameters of the 3D-printed lattice structure.

Serial Number	Lattice Cell	Unit Cell Design	Cell Parameter		CAD Construction	Specimen Edge Length (mm)
			L (mm)	D (mm)		
1	RO	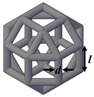	4.14	0.916	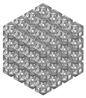	42 × 42 × 42
2	SO	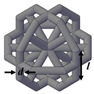	4.14	0.979	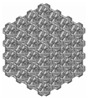	42 × 42 × 42

**Table 2:** Mineral composition of ordinary Portland cement P·O 42.5 [29]. Reproduced from Gu et al., Polymers; published by MDPI, 2025.

Composition	C_3_S	C_2_S	C_4_AF	C_3_A	F-MgO	F-CaO
Content (%)	60.5	18.1	8.9	7.4	1.8	0.9

**Table 3:** Composition of sand [29]. Reproduced from Gu et al., Polymers; published by MDPI, 2025.

SO_2_	>98%	Ignition loss	<0.47%
Moisture content	≤0.18%	Chlorine content	≤0.007%
Sediment content	≤0.18%	Other mineral content	≤0.002%

**Table 4:** The main chemical composition of fly ash particles.

Component Composition	SiO_2_	Al_2_O_3_	Fe_2_O_3_	Cao	MgO	K_2_O + Na_2_O	Ignition Loss	Others
Content (%)	52.7	25.8	9.7	3.7	1.2	2.3	0.4	4.6

**Table 5:** Performance indices of the water-reducing agent [29]. Reproduced from Gu et al., Polymers; published by MDPI, 2025.

Air content	3.4%
Moisture content	1.7%
pH (23 °C)	8.0 ± 1.0
Volume density	600 ± 100 g
Sodium sulfate content	0.79% m^3^
Cl^−^ content	0.049%
Total alkalinity	0.77%
Powder color	White
Water reduction rate of cement	≥30%
Mortar recommended dosage	0.15–0.3

**Table 6:** Maximum strain values of the specimen at different times.

Specimen	Cycle Number	Time (s)	Strain
Control	45	140	0.00082
		170	0.00465
		180	0.011
	90	140	0.00755
		170	0.0308
		180	0.0382
	145	140	0.000315
		170	0.029
		180	0.224
RO	45	160	0.03
		190	0.0412
		210	0.0442
	90	160	0.000395
		190	0.00043
		210	0.0515
	145	160	0.054
		190	0.0438
		210	0.0545
SO	45	160	0.0158
		190	0.0382
		210	0.0525
	90	160	0.03
		190	0.0472
		210	0.09
	145	160	0.0805
		190	0.055
		210	0.067

**Table 7:** Failure morphology of cement-based samples reinforced by different lattices.

Specimen	45 Cycles	90 Cycles	145 Cycles
Control	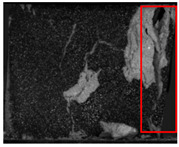	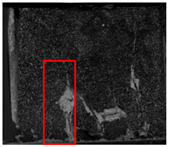	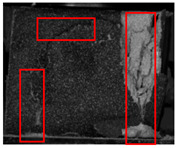
	(**a**)	(**b**)	(**c**)
RO	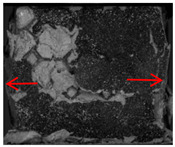	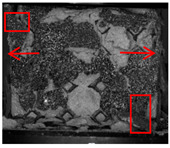	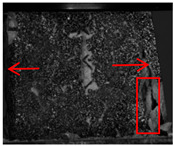
	(**d**)	(**e**)	(**f**)
SO	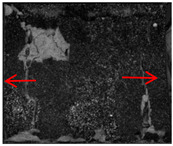	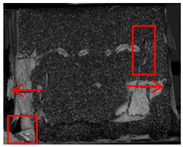	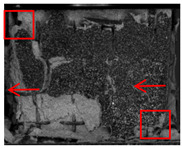
	(**g**)	(**h**)	(**i**)

## Data Availability

The original contributions presented in this study are included in the article. Further inquiries can be directed to the corresponding author.
